# Spatiotemporal Structure and Dynamics of Spontaneous Oscillatory Synchrony in the Vagal Complex

**DOI:** 10.3389/fnins.2018.00978

**Published:** 2018-12-18

**Authors:** Yoshinori Kawai

**Affiliations:** ^1^Department of Anatomy, The Jikei University School of Medicine, Tokyo, Japan; ^2^Center for Neuroscience of Pain, The Jikei University School of Medicine, Tokyo, Japan

**Keywords:** brain wave, emergence, oscillator, electrical activity, viscerosensory, self-organization

## Abstract

Fundamental structure and dynamics of spontaneous neuronal activities without apparent peripheral inputs were analyzed in the vagal complex (VC), whose activities had been generally thought to be produced almost passively to peripheral cues. The analysis included the caudal nucleus of the tractus solitarius—a main gateway for viscerosensory peripheral afferents and involved dynamically and critically in cardiorespiratory brainstem networks. In the present study, a possibility of self-organized brain activity was addressed in the VC. While VC neurons exhibited sparse firing in anesthetized rats and in *in vitro* preparations, we identified peculiar features of the emergent electrical population activity: (1) Spontaneous neuronal activity, in most cases, comprised both respiration and cardiac cycle components. (2) Population potentials of polyphasic high amplitudes reaching several millivolts emerged in synchrony with the inspiratory phase of respiratory cycles and exhibited several other characteristic temporal dynamics. (3) The spatiotemporal dynamics of local field potentials (LFPs), recorded simultaneously over multiple sites, were characterized by a stochastic emergence of high-amplitude synchrony. By adjusting amplitude and frequency (phase) over both space and time, the traveling synchrony exhibited varied degrees of coherence and power with a fluctuating balance between mutual oscillators of respiratory and cardiac frequency ranges. Full-fledged large-scale oscillatory synchrony over a wide region of the VC emerged after achieving a maximal stable balance between the two oscillators. Distinct somatic (respiratory; ~1 Hz) and visceral (autonomic; ~5 Hz) oscillators seemed to exist and communicate co-operatively in the brainstem network. Fluctuating oscillatory coupling may reflect varied degrees of synchrony influenced by the varied amplitude and frequency of neuronal activity in the VC. Intranuclear micro-, intrabulbar meso-, and wide-ranging macro-circuits involving the VC are likely to form nested networks and strategically interact to maintain a malleable whole-body homeostasis. These two brainstem oscillators could orchestrate neuronal activities of the VC, and other neuronal groups, through a phase-phase coupling mechanism to perform specific physiological functions.

## Introduction

Distinct synchronous rhythmic activities have been recorded in several peripheral nerves—notably respiratory activities for the phrenic nerves and sympatho-excitatory (cardiac) activities for sympathetic nerves, such as the splanchnic nerves (Zhong et al., 1997). These respiratory and autonomic activities are thought to exist separately in their respective nerves while exhibiting reciprocal interactions. Therefore, cardiorespiratory coupling at an individual level has been extensively investigated in animals and humans (Coleman, 1920; Dick et al., 2014). Cardiorespiratory peripheral neural activities originate in the brainstem and spinal neuronal circuits at the level of central pattern generators (Feldman and Ellenberger, 1988; Smith et al., 2007). Neuronal activities of several medullary groups of neurons including those in the rostral ventrolateral medulla (RVLM; as a sympatho-excitatory center) reportedly comprise components of both respiratory and cardiac rhythms (Boczek-Funcke et al., 1992; Habler and Janig, 1995; Pilowsky, 1995; Ootsuka et al., 2002). As a consequence, a theory of coupled oscillators for the two separate but intimately related systems, has been proposed and discussed for both the peripheral and central nervous systems.

The vagal complex (VC) consists of the caudal nucleus of the tractus solitarius (cNTS) and the dorsal motor nucleus of the vagus nerve (dmnX). The cNTS receives peripheral afferents from nerves related to cardiorespiratory regulations via pulmonary stretch and chemo- and baro-reflexes. The VC thus dynamically and strategically interacts with cardiorespiratory rhythmogenetic circuits in the brainstem (Lambertz et al., 1993; Zoccal et al., 2014). Therefore, in addition to peripherally transmitted cardiorespiratory rhythmic cues, VC neurons could exhibit centrally driven oscillatory activity. In fact, synchronized oscillations in the solitary complex of rat *in vitro* slice preparations have been reported (Fortin et al., 1992). However, surprisingly, similar rhythmic activities relating to cardiorespiratory cycles have not been reported in the VC except in rat and cat pump cells (Ezure and Tanaka, 1996; Miyazaki et al., 1998, 1999) and cat dorsal respiratory neurons (Bianchi et al., 1995). Since pump cells seem to involve a peripherally-driven neuronal activity and dorsal respiratory neurons are lacking in rats, rhythmic neuronal activities of central origin have not been investigated in the VC.

In the present study, we addressed, for the first time, whether spontaneous oscillatory activities, mainly originating from central neuronal circuits (not peripherally-driven), can be recorded in the rat VC; we also provide the first investigation into the fundamental structure and dynamics of these activities and how these activities are related to cardiorespiratory cycles both in *in vivo* and *in vitro* VC preparations. Results of the present study described several unique features of spontaneous oscillatory synchrony in the VC. Our hypothesis is that not only VC neurons but also more wide-ranging brainstem neuronal populations could exhibit a self-organizing oscillatory synchrony to maintain a malleable whole-body homeostasis.

## Materials and Methods

### Animal Preparation

All surgical and experimental procedures were approved by the Institutional Committee for the Care and Use of Experimental Animals at the Jikei University School of Medicine in Japan and were performed in accordance with the Guidelines for Proper Conduct of the Animal Experiments by the Science Council of Japan. *In vivo* electrophysiological recordings were carried out using 16 male Sprague–Dawley rats (weight range, 280–310 g). Animals were anesthetized with an intraperitoneal (i.p.) injection of ketamine (30 mg/kg) and xylazine (24 mg/kg) and placed in a stereotaxic instrument for recording. In most cases, 0.5% isoflurane was additionally administered through a nose mask to obtain sufficient depth of anesthesia during recordings.

Three rats received baro- and chemo-receptor denervation by bilateral sectioning of the carotid sinus, aortic depressor, and vagus nerves, before recordings.

*In vitro* experiments were performed on newborn Sprague–Dawley rats (P2–3, *n* = 3). Rats were anesthetized with urethane (1 g/kg, i.p.) and sacrificed by decapitation. Brainstem blocks containing medulla oblongata and pons were prepared and superfused with Krebs saline (in mM: 125 NaCl, 2.5 KCl, 2 CaCl_2_, 1 MgCl_2_, 1.25 NaH_2_PO_4_, 26 NaHCO_3_, 10 glucose) continuously bubbled with a 95% O_2_ and 5% CO_2_ gas mixture in a recording chamber for the simultaneous recording of neuronal activity in the VC and respiratory rhythmic neural activity in hypoglossal nerve rootlets.

### Electrodes and Recordings

#### *In vivo* Recordings

Glass electrodes [1.5 mm outer diameter (O.D.), World Precision Instruments, Sarasota, FL] containing 2 M NaCl were used in *in vivo* extracellular recordings. The resistance of the electrodes filled with this solution ranged from 1 to 5 MΩ. After making an incision in the atlanto-occipital dural membrane, an electrode tip was advanced vertically with a motorized micromanipulator (IVM Single, Scientifica, East Sussex, UK) into the exposed left dorsal medulla at the level of the area postrema, under a stereoscopic microscope; the depth was 50–500 μm from the brain surface. Neuronal signals were recorded in alternating current (AC) mode (Multiclamp700A, Axon Instruments, Union City, CA). The amplified signals were analyzed offline using Spike2 (Cambridge Electronic Design Limited, Cambridge, UK) and OriginPro2017 (Lightstone co., Tokyo, Japan) software.

Simultaneous 16-channel *in vivo* recordings were generated from the VC using a silicon probe (A1x16-Poly2s-5mm-50s-177-A16, NeuroNexus Technologies, Inc., Ann Arbor, MI). The resistance specified by the manufacturer was between 0.96 and 1.17 MΩ. Each electrode “site” consisted of circular platinum metal 15 μm in diameter, arranged by two 8-site-columns, and separated by 50 μm (Blanche et al., 2005). Electrical activities were amplified (A-M Systems Model 3600 Amplifier, Carlsborg, WA, USA), sampled at 1–4 kHz, and stored for offline analyses.

Cardiorespiratory activities were recorded non-invasively with a piezoelectric pulse transducer (PZT; MP100, AD Instruments, New South Wales, Australia). The PZT transformed mechanical movements or thorax vibrations (through touch on the sensor probe patch) into electrical signals that could be divided into heartbeat and respiration components (Sato et al., 2006).

#### *In vitro* Recordings

**Figure 1:**
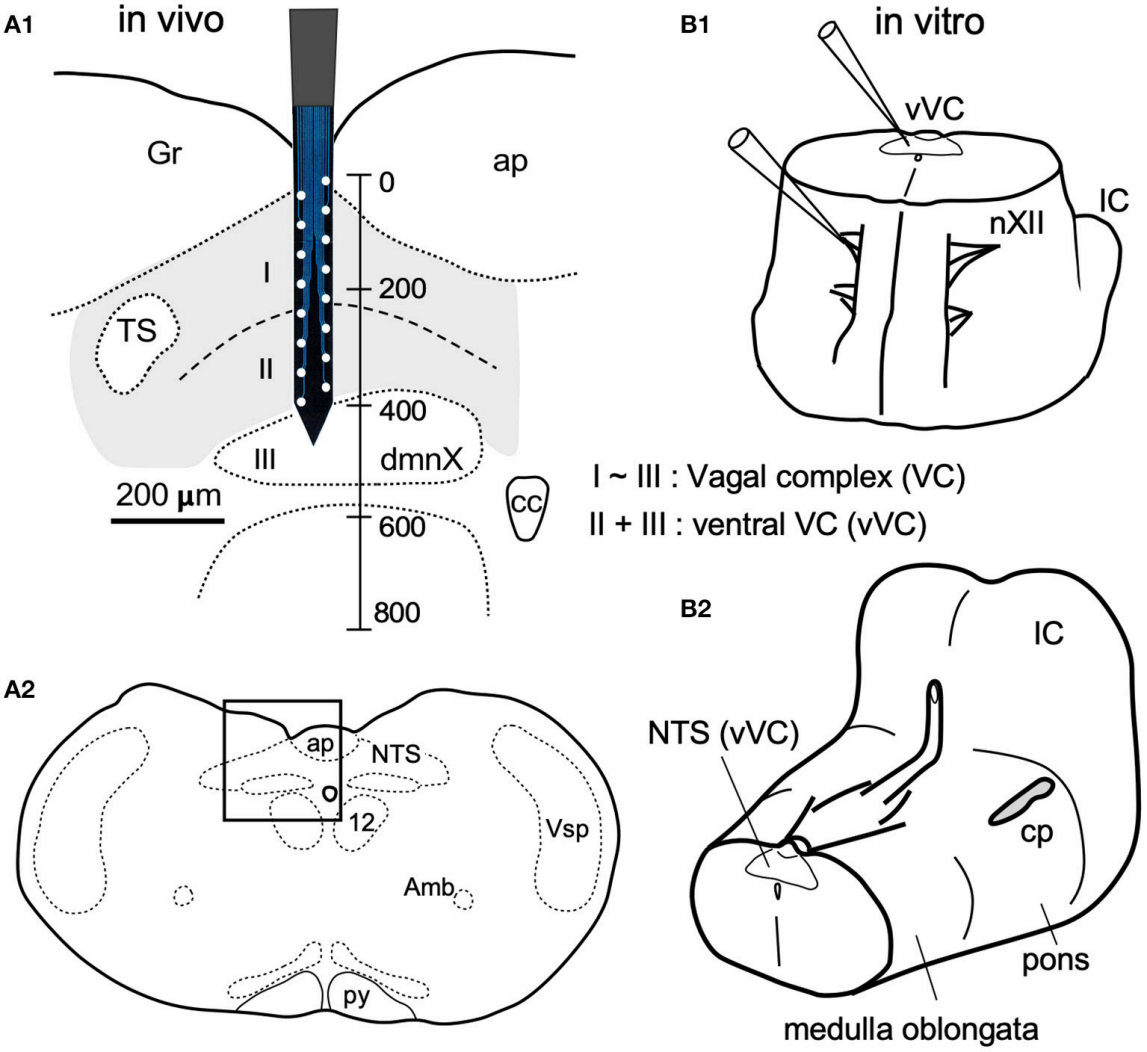
*In vivo* **(A)** and *in vitro* **(B)** preparations for recordings of spontaneous neuronal activities from the vagal complex (VC). **(A)** The VC consists of the dorsal motor nucleus of the vagus (dmnX, layer III) and the nucleus of tractus solitarius (NTS, gray-shaded) that can be divided into dorsal and ventral (I and II) layers according to function and cytoarchitecture (Negishi and Kawai, 2011). A silicon electrode of 16 metal probes spanned the whole depth of the VC (a vertical scale in μm). **(A1)** is magnified from a frame in **(A2)**. **(B1)** An upright ponto-medulla block preparation of newborn rats. **(B2)** Cerebellum was detached from this preparation. Amb, ambiguus nucleus; ap, area postrema; cc, central canal; cp, cerebellar peduncle (cut); Gr, gracilis nucleus; IC, inferior colliculus; nXII (12), hypoglossal nucleus; py, pyramidal tract; Vsp, spinal nucleus of the trigeminal nerves.

The brainstem block was set upright in a recording chamber for simultaneous recordings of VC neuronal and hypoglossal nerve activity (Figure 1). Neuronal activity was recorded by a glass electrode (1.0 mm O.D. thick-wall type, World Precision Instruments, Sarasota, FL) with a tip resistance of 10–20 MΩ (filled with 2 M potassium acetate), under a direct visual inspection of an electrode tip and neuronal somas. The hypoglossal nerve rootlets were suctioned through a glass electrode and the nerve signals were recorded simultaneously along with VC neuronal activity, in AC mode, with a Multiclamp700A amplifier.

### Data Analysis

Neuronal signals recorded *in vivo*, to a highly various degree, exhibited a mixture of single- or multi-unit spikes and local field potentials (LFPs), especially in standard glass electrodes, while signals recorded with a silicon probe mostly consisted of LFPs. For 0–10 Hz phase (cardiorespiratory rhythmic frequency range) enhancement or extraction, neuronal signals were, in some cases, offline filtered with a low-pass type II Chebyshev filter (Spike2, low-filtered between D.C. and 100 Hz with an order of 2 and a ripple of 60).

Cross- and auto-correlograms, fast Fourier transform (FFT) power and coherence spectra, and continuous wavelet transform (CWT, Morlet wavelets; each with 5 cycles) were performed with OriginPro2017. Wavelet coherence was performed using Morse wavelets (default wavelet function) with MATLAB (The MathWorks, Natick, MA). Wavelet analyses (CWT and wavelet coherence) were performed on PZT and low-pass filtered neuronal signals. CWT and wavelet coherence were expressed as time-resolved power and coherence spectra, respectively.

Cross-correlations are linear estimators to measure temporal variations of coherence of a signal. They were computed according to the relation:

$$\begin{array}{l} C_{ij}\left(\tau \right)=\frac{\sum_{k}\nu \left(r_{i}{,t}_{k}\right)\nu \left(r_{j},t_{k}+\tau \right)}{{\sum_{k}\nu \left(r_{i},t_{k}\right)}^{2}} \end{array}$$

where the correlation expresses the average of the normalized LFP ν(*r*_*i*_, *t*_*j*_) at site *r*_*i*_ and time *t*_*j*_, multiplied by the normalized LFP ν(*r*_*j*_, *t*_*k*_ + τ) at site *r*_*j*_ and time *t*_*k*_ + τ. *C*_*ij*_(τ) varies between −1 and +1. Efficient algorithms based on FFTs were used to evaluate *C*_*ij*_(τ). The auto-correlation *C*_*ij*_(τ) is obtained by setting *i* = *j*. *C*_*ij*_(τ) measures how a signal is temporally coherent with itself: its value stays close to unity as long as the signal is correlated; it oscillates for periodic oscillations and decays toward zero for irregular signals (Destexhe et al., 1999).

The coherence spectrum of two time series, *x* and *y*, is:

$$\begin{array}{l} C_{xy}\left(a,b\right)=S\left(C_{x}^{*}\left(a,b\right)C_{y}\left(a,b\right)\right) \end{array}$$

Wavelet coherence is a measure of the correlation between two signals.

The wavelet coherence of two time series *x* and *y* is:

$$\begin{array}{l} \frac{|S\left(C_{x}^{*}\left(a,\;b\right)C_{y}\left(a,\;b\right)\right){|}^{2}}{S\left(|C_{x}\left(a,b\right){|}^{2}\right)S\left(|C_{y}\left(a,b\right){|}^{2}\right)} \end{array}$$

*C*_*x*_(*a, b*) and *C*_*y*_(*a, b*) denote the continuous wavelet transforms of *x* and *y* at scales *a* and positions *b*. The superscript ^*^ is the complex conjugate and *S* is a smoothing operator in time and scale.

For real-valued time series, the wavelet coherence is real-valued if a real-valued analyzing wavelet is used, and complex-valued if a complex-valued analyzing wavelet is used.

The Fourier transform of the generalized Morse wavelet is:

$$\begin{array}{l} \psi_{P,\gamma }\left(\omega \right)=U\left(\omega \right)a_{P,\gamma }\omega^{\frac{P^{2}}{\gamma }}e^{{-\omega }^{\gamma }} \end{array}$$

where *U*(ω) is the unit step, *a*_*P*, γ_ is a normalizing constant, *P* is the time-bandwidth product, and γ characterizes the symmetry of the Morse wavelet. Much of the literature about Morse wavelets uses β, which can be viewed as a decay or compactness parameter, rather than the time-bandwidth product, *P* = $\sqrt{\beta \gamma }$. The equation for the Morse wavelet in the Fourier domain parameterized by β and γ is:

$$\begin{array}{l} \psi_{\beta ,\gamma }\left(\omega \right)=U\left(\omega \right)a_{\beta ,\gamma }\omega^{\beta }e^{-\omega^{\gamma }} \end{array}$$

In the CWT, the analyzing function is a wavelet, ψ. The CWT compares the signal to shifted and compressed or stretched versions of a wavelet. Stretching or compressing a function is collectively referred to as *dilation* or *scaling* and corresponds to the physical notion of *scale*. By comparing the signal to the wavelet at various scales and positions, a function of two variables are obtained. The 2-D representation of a 1-D signal is redundant. If the wavelet is complex-valued, the CWT is a complex-valued function of scale and position. If the signal is real-valued, the CWT is a real-valued function of scale and position. For a scale parameter, a > 0, and position, *b*, the CWT is:

$$\begin{array}{l} C\left(a,b;f\left(t\right),\psi \left(t\right)\right)=\int_{-\infty }^{\infty }f\left(t\right)\frac{1}{a}f\left(t\right)\psi^{*}\left(\frac{t-b}{a}\right)dt \end{array}$$

where ^*^ denotes the complex conjugate. Not only do the values of scale and position affect the CWT coefficients, the choice of wavelet also affects the values of the coefficients.

By continuously varying the values of the scale parameter, *a*, and the position parameter, *b*, the *cwt coefficients C(a,b)* are obtained . Note that for convenience, the dependence of the CWT coefficients on the function and analyzing wavelet has been suppressed.

Morlet wavelet (five cycles) is:

$$\begin{array}{l} \psi \left(t\right)=\pi^{-\frac{1}{4}}e^{-\frac{1}{2}t^{2}}\cos\left(5t\right) \end{array}$$

(https://www.originlab.com/doc/User-Guide; https://jp.mathworks.com/help/matlab/).

For the relationship between correlogram values and amplitudes of LFPs (**Figure 8**), LFP signals were rectified and integrated with a time constant of 50–100 ms by the Spike2 Chebyshev filter. Total integrated area over a period of 10 s was obtained using OriginPro2017. The averaged area of paired signals was used for each value on the abscissa. For correlograms, according to distances between paired recording sites, means ± standard deviations (SDs) of correlations were plotted on the abscissa alongside all the used values at each distance. For both correlation graphs, each value (**Figure 8D1**) or mean value (**Figure 8D2**) was linearly fit using OriginPro2017.

## Results

The VC, consisting of the cNTS and the dmnX, is a three-layered structure (I–III) in the dorsomedial medulla oblongata (Figure 1). The depth along the dorsoventral axis of the adult rats was 400 μm. The dimensions of the silicon probe used in the present study for *in vivo* recordings is shown in Figure 1A1. For *in vitro* experiments, an electrode tip for neuronal recordings was positioned on cell somas under a direct inspection in the ventral VC (vVC; Figure 1B1, thus described since a boundary between layers II and III was often obscure).

### High-Amplitude Poly-Phasic Potentials in Anesthetized Animals

**Figure 2:**
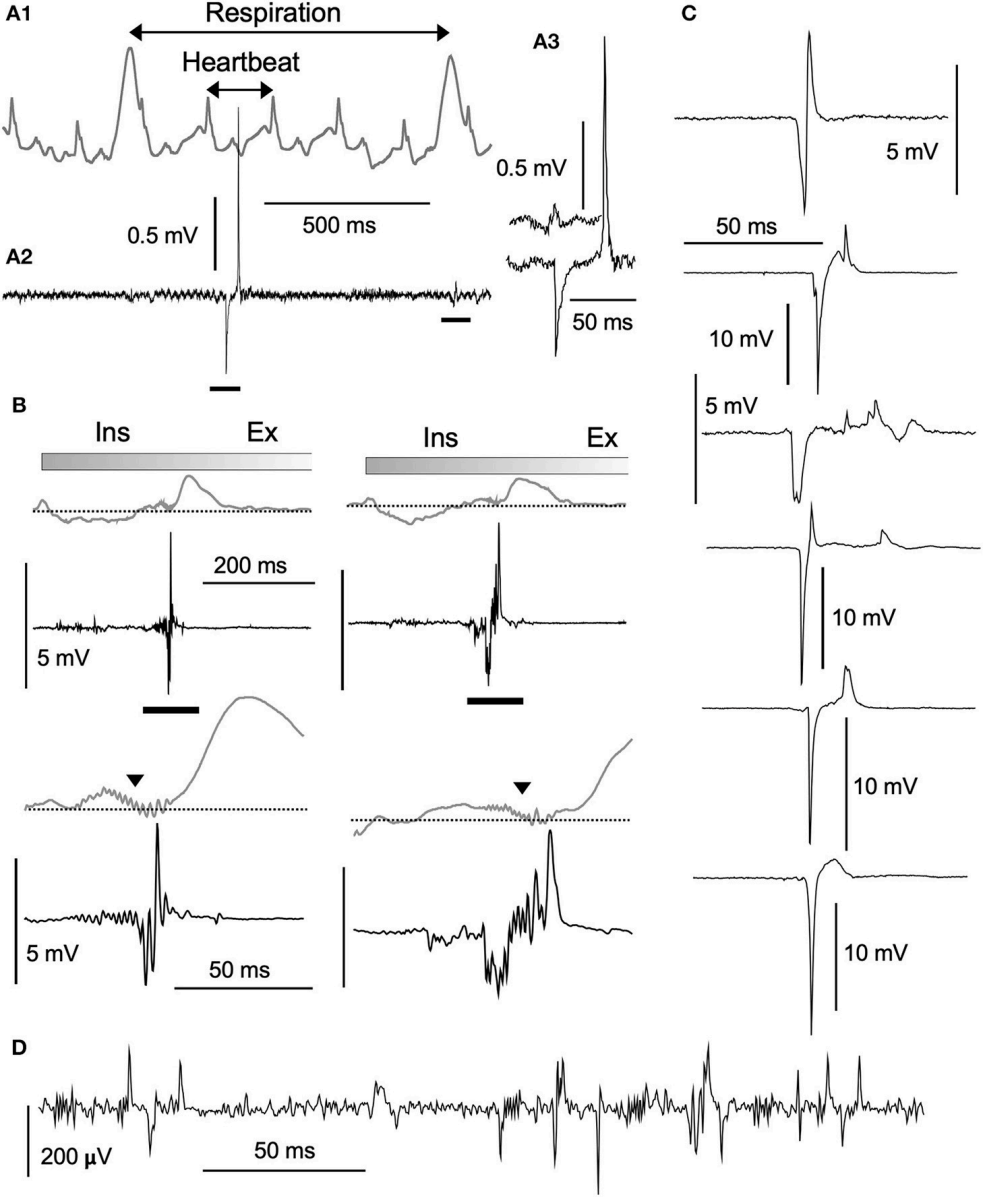
Structure of high-amplitude potentials. Neuronal high-amplitude potentials recorded extracellularly *in vivo* using a standard glass electrode in the vagal complex. **(A1)** Cardiorespiratory activities recorded simultaneously with a piezoelectric transducer (PZT) attached to a thorax (gray wave in an upper row). Cardiorespiratory cycles (“Respiration” and “Heartbeat” cycles indicated by double-headed arrows) were confirmed by a visual inspection of thorax movement. A simultaneously recorded neuronal activity (**A2**, lower row) contains a high-amplitude potential and a typical low-amplitude wave (**A3**, bars in **A2**). **(B)** Polyphasic high-amplitude potentials synchronized with the inspiratory (Ins) phase of each respiratory cycle. The polyphasic potential activity is reflected as synchronized jitter in cardiorespiratory PZT traces (triangles in lower rows expanded from bars in upper rows). Varied shapes of high-amplitude potentials ranging several millivolts in amplitude, as depicted in **(B)** (synchronized with respiratory cycles) and **(C)** (asynchronous with cardiorespiratory cycles), look like enlarged copies of typical low-amplitude potentials ranging hundred microvolts **(D)**. Burst-like polyphasic activities are associated with both types of potentials. Ex, expiratory phase of a respiratory cycle.

Based on PZT signals (Figure 2A1), respiration and heartbeat rates of ketamine/xylazine anesthetized rats were 0.96 ± 0.33 Hz (0.56–1.65) and 5.39 ± 0.73 Hz (4.19–6.97; *n* = 12), respectively. These values were calculated on 12 rats based on mean respiration and heartbeat rates over stable recordings of 100 s in each individual. Large respiration cycle signals appeared as either positive (peaks) or negative (troughs), depending on the transducer position relative to the diaphragms. Simultaneous recordings of neuronal activities with a standard glass electrode revealed highly varied types of waves in terms of amplitude and frequency (Figures 2A1–3). In most cases, spontaneous spikes were rarely recorded and longer recordings of several hours in fixed locations scarcely detected any emergent neuronal activities. In addition to typical single- and multi-unit spikes, highly poly-phasic or LFP-like longer-duration waves of several hundred microvolts in amplitude were also recorded (Figure 2D). Of these quite heterogeneous mixtures of neuronal signal forms, the most peculiar of the rarely observed waves had high amplitudes (~2 to ~15 millivolts) (Figures 2B,C). They appeared either as asynchronous (Figures 2A1,C) or synchronous (Figure 2B) waves with PZT signal peaks or troughs representing salient cardiorespiratory cycles. The most frequently-recorded high-amplitude potential was the highly-polyphasic type synchronized with an inspiration phase of respiratory cycles (Figure 2B).

### Neuronal Activities of the Vagal Complex Contain Cardiorespiratory Cycle Components

**Figure 3:**
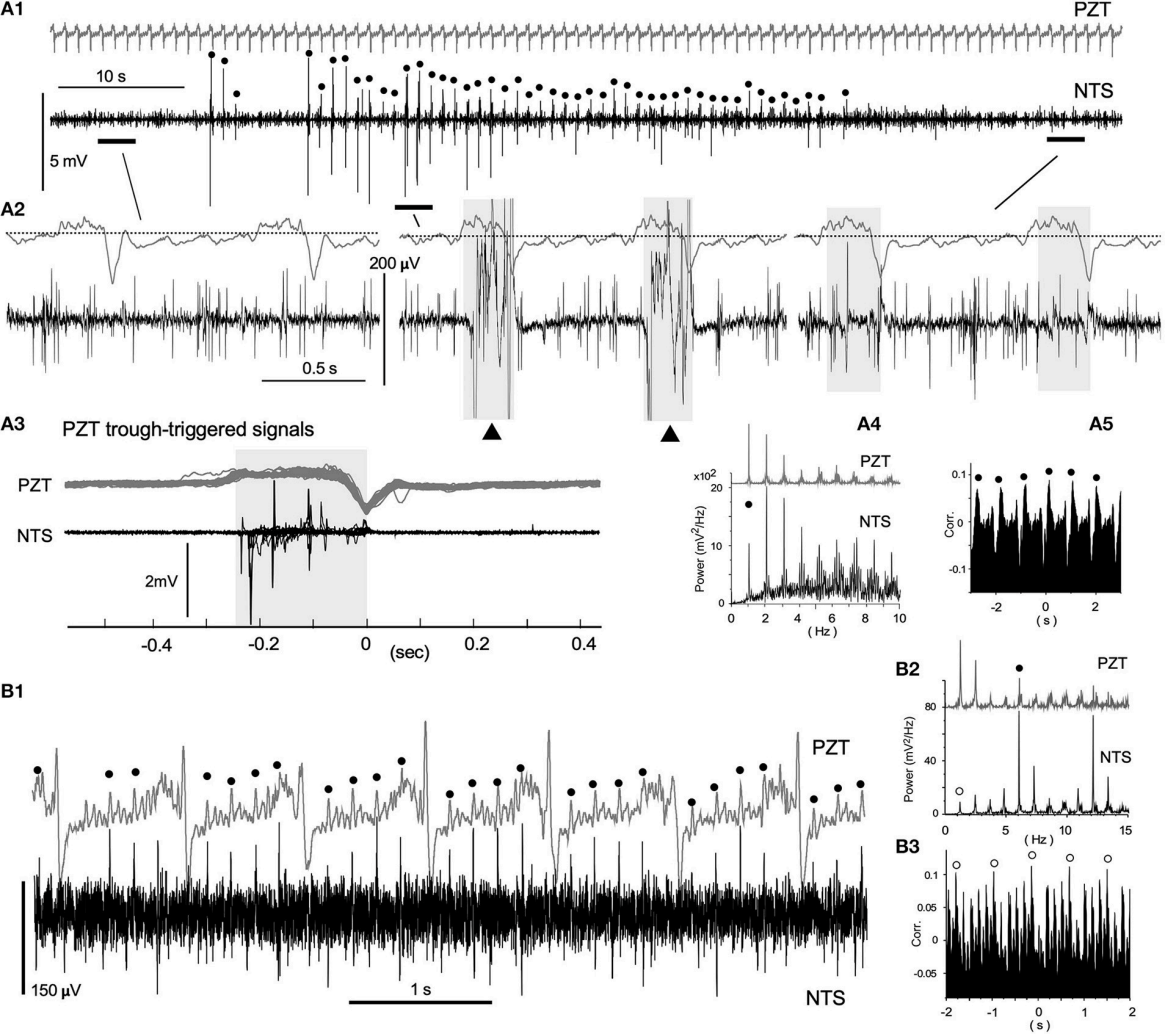
Neuronal activities synchronized with cardiorespiratory cycles, recorded *in vivo* using a glass electrode. **(A1)** Episodic emergence of trains of high-amplitude potentials are synchronized with the respiratory cycle. PZT traces (**A1–3**; shown in gray) exhibit a respiratory cycle of ~1.1 Hz and a heartbeat cycle of ~5.6 Hz. Trains of high-amplitude potentials synchronized with respiratory cycles are marked with solid dots. Three episodes of a 3–4 s duration before, during, and after the high-amplitude potential trains, corresponding to horizontal bars in **(A1)** are shown in **(A2)** with simultaneously-recorded PZT traces. High-amplitude potential traces in the middle are truncated. Note the polyphasic high-amplitude potentials (solid triangles) synchronized with the inspiration phase (gray-shaded). The last episode contains low-amplitude and longer-duration signals during the inspiration phase (gray-shaded) in addition to short-duration spikes. **(A3)** PZT-trough-triggered high-amplitude potentials of 20 successive respiratory cycles. Multiple potentials appear during the inspiration phase (gray-shaded). **(A4)** and **(A5)** Power-spectrum analyses (Power) and a correlogram (Corr.) of neuronal and cardiorespiratory (PZT: gray in **A4**) activities are shown. Respiratory peaks of ~1 Hz are marked with solid circles. **(B1)** Low-amplitude spikes synchronized with heartbeat cycles. A simultaneous *in vivo* recording of neuronal and cardiorespiratory (in gray) activities indicate synchrony of short-duration spikes of ~100 μV amplitude with heartbeat cycles of ~5.9 Hz (solid circles). **(B2)** and **(B3)**, Power-spectrum analyses (Power) and a correlogram (Corr.) of neuronal and cardiorespiratory (PZT: gray in **B2**) activities. Heartbeat and respiratory peaks are marked with solid and open circles, respectively. NTS, the nucleus tractus solitarius.

Spontaneous neuronal activities of the VC exhibited varied episodic behaviors characterized by a mixture of periodic oscillations and apparent randomness (Figure 3). Figure 3A shows an episode of a ~90 s simultaneous recording using a PZT (upper in gray) for cardiorespiratory signals and a standard glass electrode for neuronal activities. Respiratory and heartbeat frequencies judged by the PZT signals were 1.06 ± 0.02 Hz and 5.60 ± 0.19 Hz, respectively. Polyphasic high-amplitude neuronal signals coincided with PZT troughs (Figure 3A1 dots and Figure 3A2 middle). Low-amplitude (100–200 μV) spike-like signals of short-duration (~2 ms or less), asynchronous with cardiorespiratory cycles, were recorded throughout the whole episode, while some longer-duration waves (LFP-like signals) were synchronized with inspiration phases of respiratory cycles (Figures 3A2,3, shaded in gray in the middle and right short episodes). Power spectrum and correlogram analyses of the neuronal and PZT (gray) signals show coherent respiratory frequencies of ~1 Hz (dots in Figures 3A4,5). In this case of recordings, no apparent synchrony was evident between neuronal and heartbeat activities. Figure 3B shows 5.88 Hz coherence between neuronal and PZT heartbeat signals as indicated by dots. In this example, the 5.88 Hz power (dots in Figures 3B1, 2) of the neuronal signals was higher than the 1.22 Hz that corresponded to PZT respiratory signals (open circles in Figures 3B2,3). The correlogram between neuronal and PZT signals shows both respiratory (open circles) and heartbeat frequency range oscillations.

Fundamentally similar spontaneous neuronal activities were also recorded in bilateral vagatomized and baro-/chemo-receptor denervated rats. It was concluded that spontaneous neuronal activities, containing cardiorespiratory cycle components, were, for the most part, shaped by central brainstem neuronal circuits rather than the cardiorespiratory reflex of peripheral origins.

### Neuronal Activities Synchronized With Respiratory Cycles in the Vagal Complex *in vitro*

**Figure 4:**
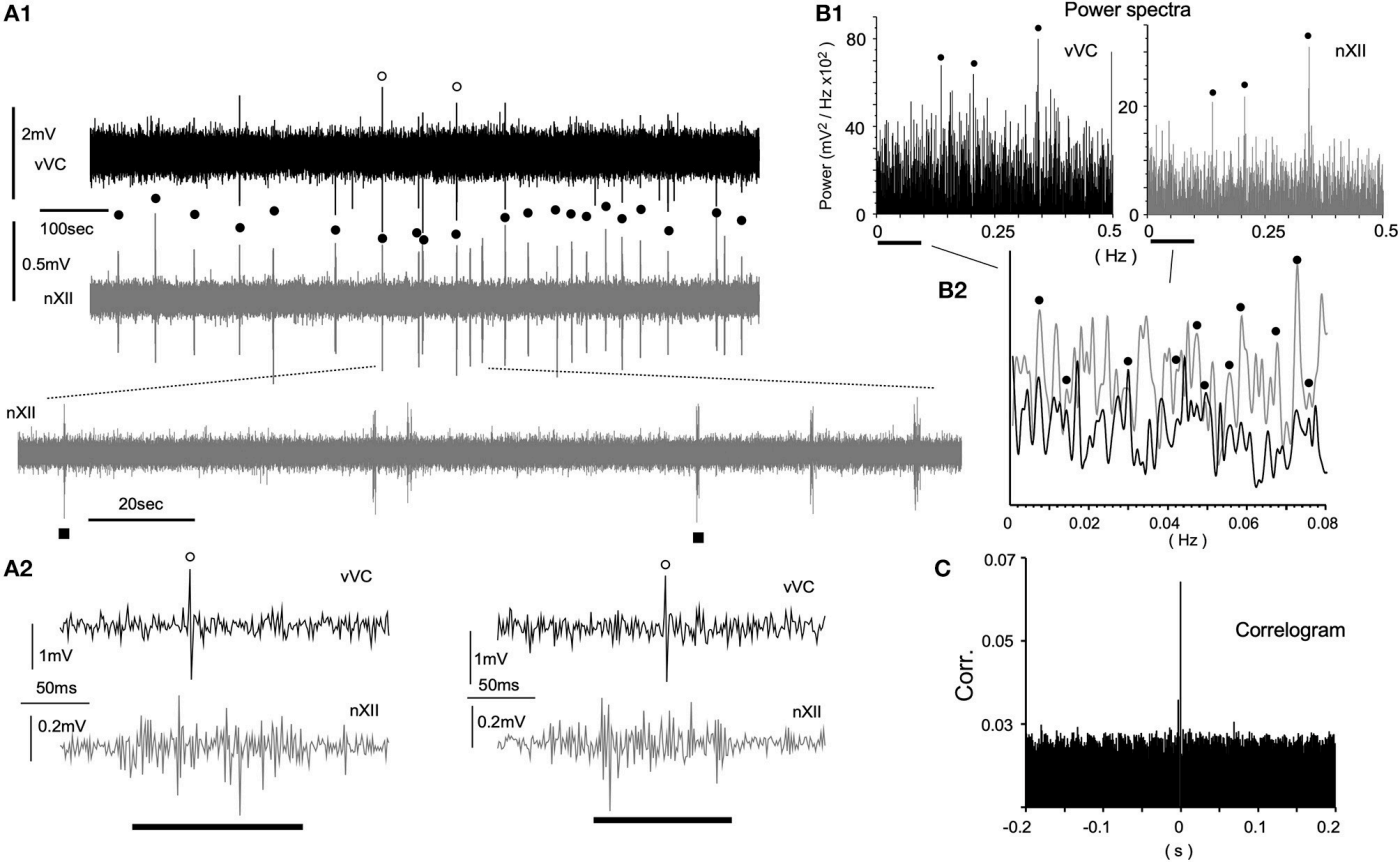
Spontaneous neuronal activities of the ventral vagal complex (vVC) synchronized with presumed respiratory rhythms recorded in an *in vitro* ponto-medullary block. **(A)** Simultaneous recordings of a vVC neuron and hypoglossal nerve rootlets (nXII; in gray) reveal synchronous activities (solid circles) between them. Expanded traces (**A1** lower and **A2**) show concurrent single (vVC) and multiple (nXII) spikes. **(B)** Power spectrum analyses (Power) of vVC and nXII (in gray) activities over slow **(B1)** and slower **(B2)** frequency ranges. A vertical axis in **(B2)** is arbitrarily set for a comparison of peaks of the two sets of power. Synchronous peaks of power spectra are marked by solid circles **(B1,2)**. **(C)** A correlogram (Corr.) of vVC and nXII activities.

Since spontaneous spiking was rarely recorded in the VC *in vivo*, it was expected that spontaneous spiking would more infrequently occur *in vitro*. To increase the chance of recording a spike, we attempted a direct visualization of both the neuronal soma and the electrode tip, while simultaneously recording signals from hypoglossal nerve rootlets (nXII) suctioned into a tight glass electrode. Figure 4 shows a simultaneous recording from vVC neurons and the nXII signals. Neuronal activities were recorded as sporadic spiking units, whereas nXII signals were recorded as polyphasic bursting potentials, which varied by several 10 s intervals (Figures 4A1,2). Most of the spikes recorded in the vVC were synchronized with the nXII polyphasic signals (Figures 4A1, 2). Power spectrum analysis indicated several peaks of coherence over a slower frequency range (0.005–0.4 Hz, Figures 4B1,2). A non-periodic correlation of small values between neuronal and nerve signals was confirmed by the correlogram (Figure 4C).

### Temporal Phase Transition of Neuronal Activities in Synchrony With Cardiorespiratory Cycles

**Figure 5:**
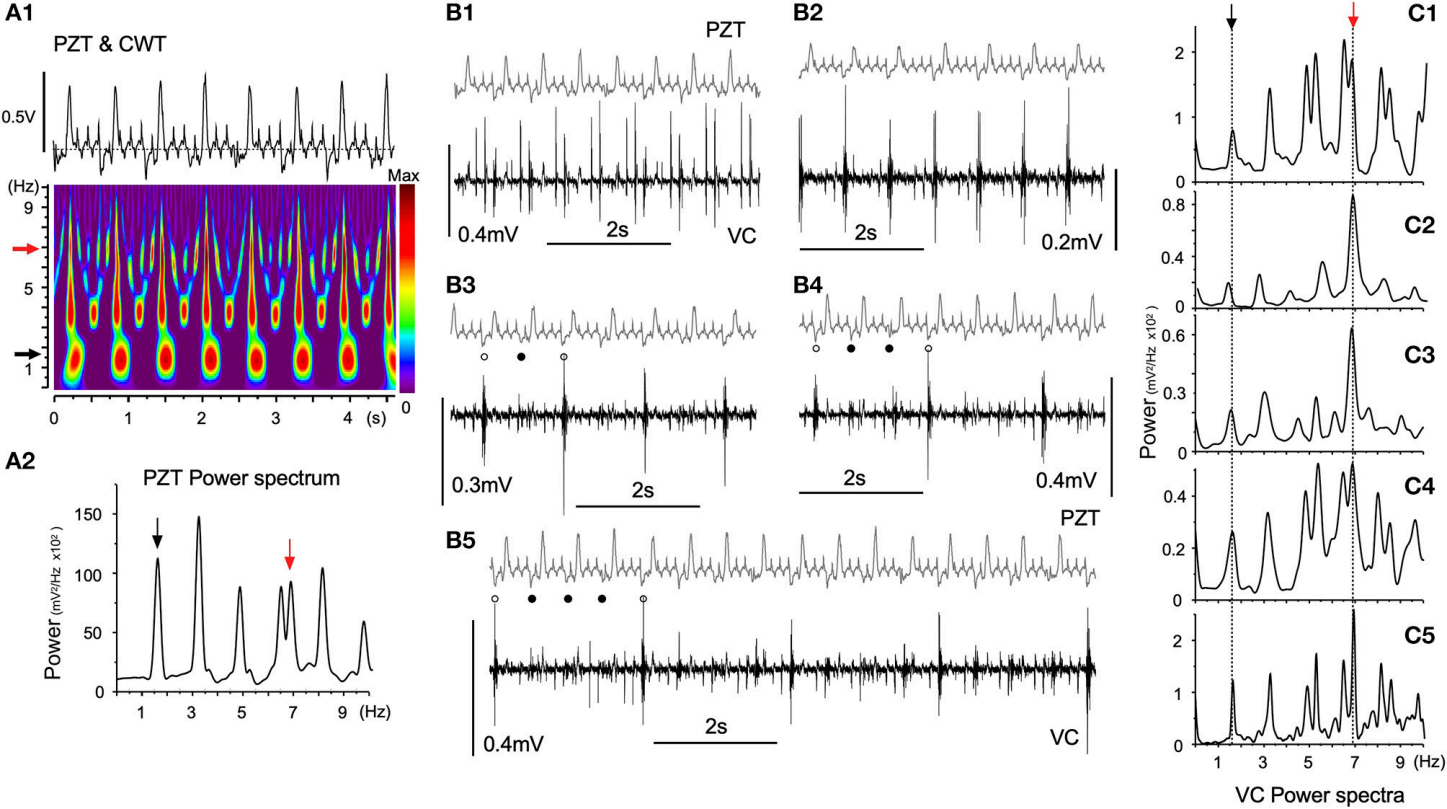
Temporal dynamics of vagal complex (VC) neuronal activities synchronized with cardiorespiratory rhythms. **(A)** A piezoelectric pulse transducer (PZT) recorded cardiorespiratory rhythmic wave and the time-resolved power spectrum by the continuous wavelet transform (CWT) analysis **(A1)**. Respiratory (1.65 Hz, black arrow) and cardiac (6.97 Hz, red arrow) rhythms are shown in the CWT time-resolved power spectrum. **(A2)** An FFT power spectrum of PZT consists of fundamental respiratory (1.65 Hz, black arrow) and cardiac (6.97 Hz, red arrow) frequency peaks and several respiratory harmonic peaks. **(B)** Temporal phase transition of polyphasic high-amplitude potentials occurring in synchrony with every cardiac cycle **(B1)**, every respiratory cycle **(B2)**, every second respiratory cycle (**B3**, open and solid dots), every third respiratory cycle (**B4**, open and solid dots), and every fourth respiratory cycle (**B5**, open and solid dots). **(C)** Ten-s-duration power spectra **(C1–5)** corresponding to **(B1–5)** recordings, respectively. Arrows (black and red) indicate respiratory and cardiac frequency components corresponding to those in PZT **(A2)**.

Figure 5 shows episodes (each for several seconds) of a simultaneous recording of PZT signals and neuronal activities from a fixed site in the VC over several hours using a glass electrode. Respiratory and heartbeat frequencies judged by PZT signals were 1.65 ± 0.04 Hz and 6.97 ± 0.32 Hz, respectively (Figure 5A1). The cardiorespiratory cycle frequencies were confirmed by time-resolved and 10-s-duration power spectrum analyses (Figures 5A1,2, black and red arrows, respectively). The time-resolved power spectrum results show intense signal spots of ~1.7 and ~3.4 Hz respiration-related frequencies (Figure 5A1) with whole number harmonics of the respiratory fundamental frequency. Neuronal signals showed varied but regular temporal patterns with respect to the frequency of polyphasic high-amplitude potentials (Figure 5B). The interval of polyphasic high-amplitude potentials was a whole number (2, 3, and 4:1) with respect to the fundamental respiratory cycle frequency of ~1.7 Hz (Figures 5B2–5). In some occasions, the interval of high-amplitude potentials was in synchrony with the heartbeat cycle (Figure 5B1). Given that the ratio of heart beats to breaths was often a whole number (4:1) in this recording as well, a preference for whole number ratios for the temporal phase transition of VC neuronal activity was noted, as seen in similar 10-s-duration power spectrum analyses (Figures 5A2,C1–5).

It was concluded that neuronal activities of the VC could exhibit fundamentally oscillational behaviors, showing patterned temporal dynamics, while keeping in synchrony with cardiorespiratory rhythms. Given that each neuronal activity was in synchrony with cardiorespiratory rhythms, it was expected that large-scale LFPs would exhibit an oscillatory synchrony with specific spatiotemporal dynamics. To analyze possible spatiotemporal dynamics, large-scale multiple recordings were performed.

### Local and Large-Scale Coherence of Local Field Potentials

**Figure 6:**
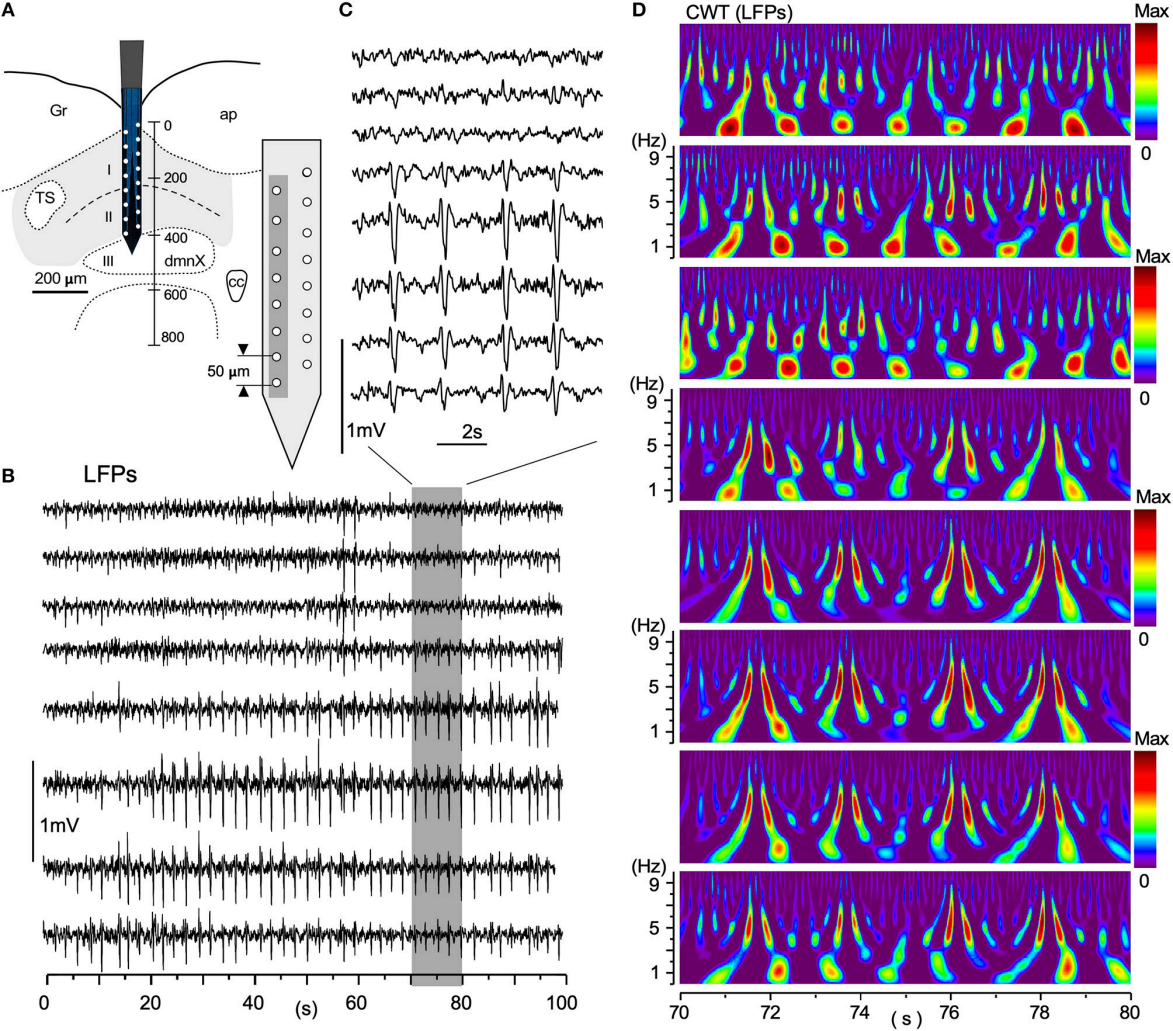
A simultaneous multiple recording of local field potentials (LFPs). **(A)** A two-row silicon probe consisting of 16 electrodes each separated by 50 μm in length and 400 μm in depth, in the vagal complex (VC). **(B)** An example of LFPs of 100 s duration from eight vertically arranged sites (gray in **A**). **(C)** An expanded 10 s-duration-LFPs from a period of 70–80 s in **(B)** (gray rectangle). **(D)** CWT time-resolved power spectrum results corresponding to this 10-s period of simultaneous recordings. Note that lower-amplitude LFPs in the dorsal VC have more intense signals of respiratory frequency range (~1 Hz) and higher-amplitude signals in the ventral cardiac range (~5 Hz). The spatial wave structure shaped by differential frequency ranges fluctuates temporally over 100 s and higher-amplitude waves are noted in deeper layers (II and III) **(B)**. CWT, continuous wavelet transform.

Large-scale neuronal signals recorded by a silicon multiple electrode revealed uneven spatiotemporal activity in terms of wave phase, amplitude, and degrees of synchrony (Figure 6). The distance between adjacent recording sites was 50 μm (Figure 6A, gray-shaded rectangle). Figure 6B shows an example of simultaneous LFPs lasting 100 s recorded from eight sites vertically across the VC. A gray-rectangle depicting a 10 s duration recording (Figure 6B; 70–80 s), expanded in Figure 6C, shows an apparent synchrony of large-amplitude LFPs of ~0.5 Hz intervals in the deeper VC. Large-scale LFP signals have lower amplitudes in the superficial layer (I in Figure 6A) of the VC, while the amplitudes are high in deeper layers (II and III). The corresponding time-resolved power spectrum results in Figure 6D show that intense signals of a large circular shape, the center of which is positioned at every ~1 Hz, appear at ~1 Hz intervals in the more dorsal VC, while pairs of vertically-long higher signals ranging from 3 to 7 Hz correspond to high-amplitude potentials at ~0.5 Hz intervals in the more ventral VC (Figure 6C). These neuronal signal frequency ranges corresponded with cardiorespiratory rhythms.

### Spatiotemporal Dynamics of Correlation and Coherence

**Figure 7:**
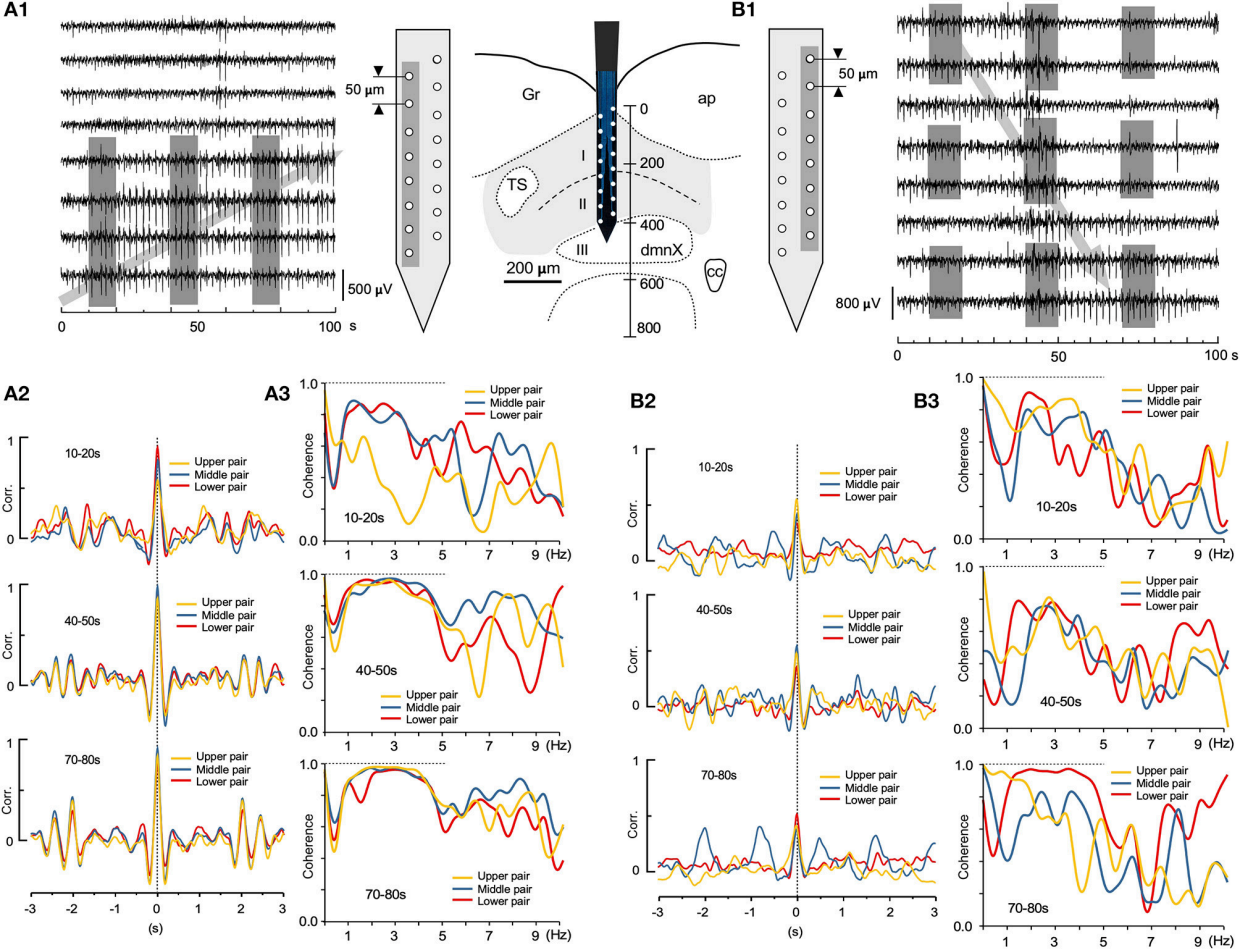
Spatiotemporal dynamics of wave correlation (Corr.) and coherence of multiple local field potentials (LFPs). Wave Corr. and coherence of a 10 s duration between LFPs recorded 30 s apart from neighboring pairs of electrodes (gray shades in **A1** and **B1**) across the depth of the vagal complex. Note a tendency of temporally-upward (gray arrow in **A1**) or -downward (gray arrow in **B1**) increases in Corr. (3 colored pairs in **A2,B2**) and an apparent higher coherence during 1–5 Hz frequency range (3 colored pairs in **A3,B3**).

Two sets of large-scale recordings from the VC seem to indicate that the fluctuating assembly of larger-amplitude waves travel either in an ascending or descending direction (Figures 7A1,B1; gray arrows, respectively), while synchronizing to neighboring waves. Correlation and coherence, evaluated every 30 s, at three successive 10 s windows, from pairs of adjacent recording sites (gray-shaded in Figures 7A1,B1), showed that fluctuating assemblies of high-amplitude waves had a higher correlation over a slower oscillation (0.2–1 Hz; Figures 7A2,B2) and a higher coherence over a frequency range of 1–5 Hz (Figures 7A3,B3), indicating possible spatiotemporal dynamics of oscillatory synchrony by activated neuronal assembly. These examples of spatially-uneven moderate coherence of higher-amplitude waves seemed to exhibit fluctuating episodic transitions from lower correlated sets of lower-amplitude waves.

### Amplitude and Distance of Paired Local Field Potentials in Relation to Degrees of Correlation

**Figure 8:**
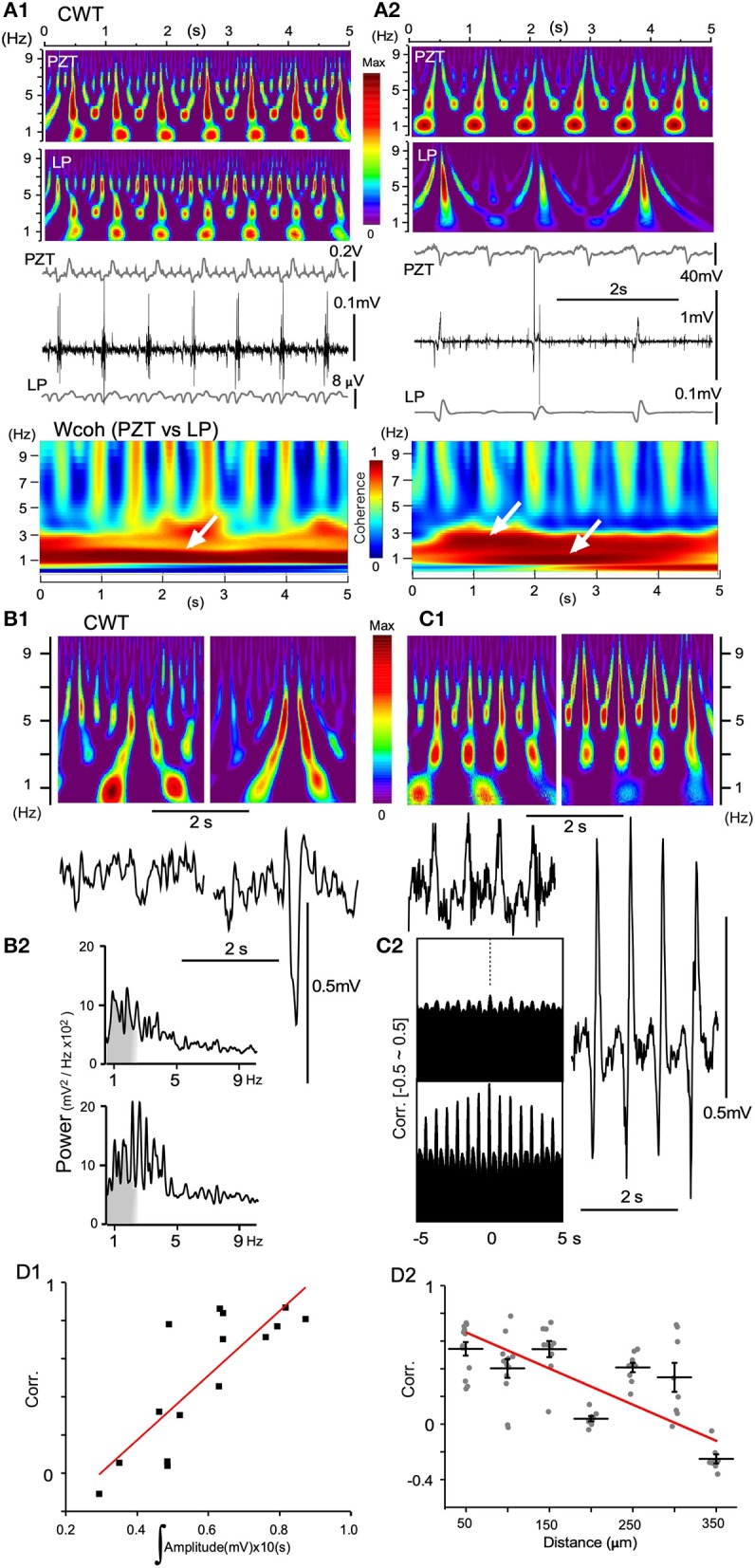
Continuous wavelet transform (CWT) time-resolved power spectrum profiles predict signal amplitude and frequency. **(A)** Dynamics of vagal complex (VC) local field potentials (LFPs) obtained offline by digitally-low pass filtering (LP) in synchrony with piezoelectric pulse transducer (PZT) signals. **(A1)** LP signals (gray in the lowest trace) obtained from original high-amplitude potentials in synchrony with every inspiration phase (middle traces) and PZT signals (gray in the upper trace) produce similar CWT structures. **(A2)** LP and PZT signals give different CWT profiles. Compared to PZT, the LP CWT profile has intense signals for larger frequencies (> ~5 Hz) and very low signals for ~1 Hz frequencies. Note that amplitudes of LP signals are far larger than those shown in **(A1)**, and the VC high-amplitude signals synchronize with every second respiratory component of the PZT signals. Time-resolved coherence spectra (wavelet coherence; Wcoh) between PZT and LP signals show different profiles with either ~1 Hz or ~1/~3 Hz dense bands (white arrows). **(B)** and **(C)**, CWT profiles (upper in **B1,C1**) and the corresponding VC LFPs (lower in **B1,C1**) recorded with a multiple silicon electrode. **(B1)** Two episodes of recordings from the same site temporally 5 s apart. **(B2)** Power spectrum measurements of each episode over a period of ~5 s. **(C1)** Two simultaneous recordings from two sites spatially 300 μm apart. **(C2)** Correlograms (Corr.) of each episode between adjacent LFPs (50 μm apart). **(D1)** and **(D2)** Linear correlation between Corr. of two simultaneously-recorded LFPs according to the mean signal amplitudes **(D1)** and the distances between recording sites **(D2)**.

LFPs obtained from recordings with either single glass electrodes or silicon probes showed similar characteristic profiles of time-scaled power spectrum results compared with those of PZT. When the amplitude of a low-pass filtered signal (LP in Figure 8A) or LFP was small, the spectral profile of the signal was very similar to that of a PZT (Figures 8A1,B1,C1) in that a ~1 Hz signal corresponding to a respiratory fundamental rhythm was conspicuous compared to a higher frequency-ranged (3–7 Hz) signal. This is also confirmed in wavelet coherence (Wcoh) profiles (Figure 8A). In contrast, as wave amplitudes grew larger, ~1 Hz signals became weaker, while 3–7 Hz range signals grew more intense (Figures 8A2,B1,C1). Spatiotemporal relationships between LFP amplitudes and the time-resolved spectral profiles were confirmed and reflected in absolute power spectrum results (Figure 8B2) and cross-correlation analyses (Figure 8C2). A rapid power shift is evident from ~1 Hz to a higher frequency (Figure 8B2). The correlogram indicates that correlation is high and oscillation is conspicuous for the higher-amplitude wave pair (Figure 8C2). The correlogram for amplitude area was produced by evaluating 16 paired LFPs (10 s windows). For the distance correlogram, 7–10 paired LFPs (10 s windows) for each distance (50–350 μm) were evaluated. A quantitative evaluation of the relationship showed a positive linearity between degrees of correlation and amplitude of paired LFPs, while a negative linearity between degrees of correlation and distance of paired LFPs (Figure 8D).

### Large-Scale Oscillatory Synchrony

**Figure 9:**
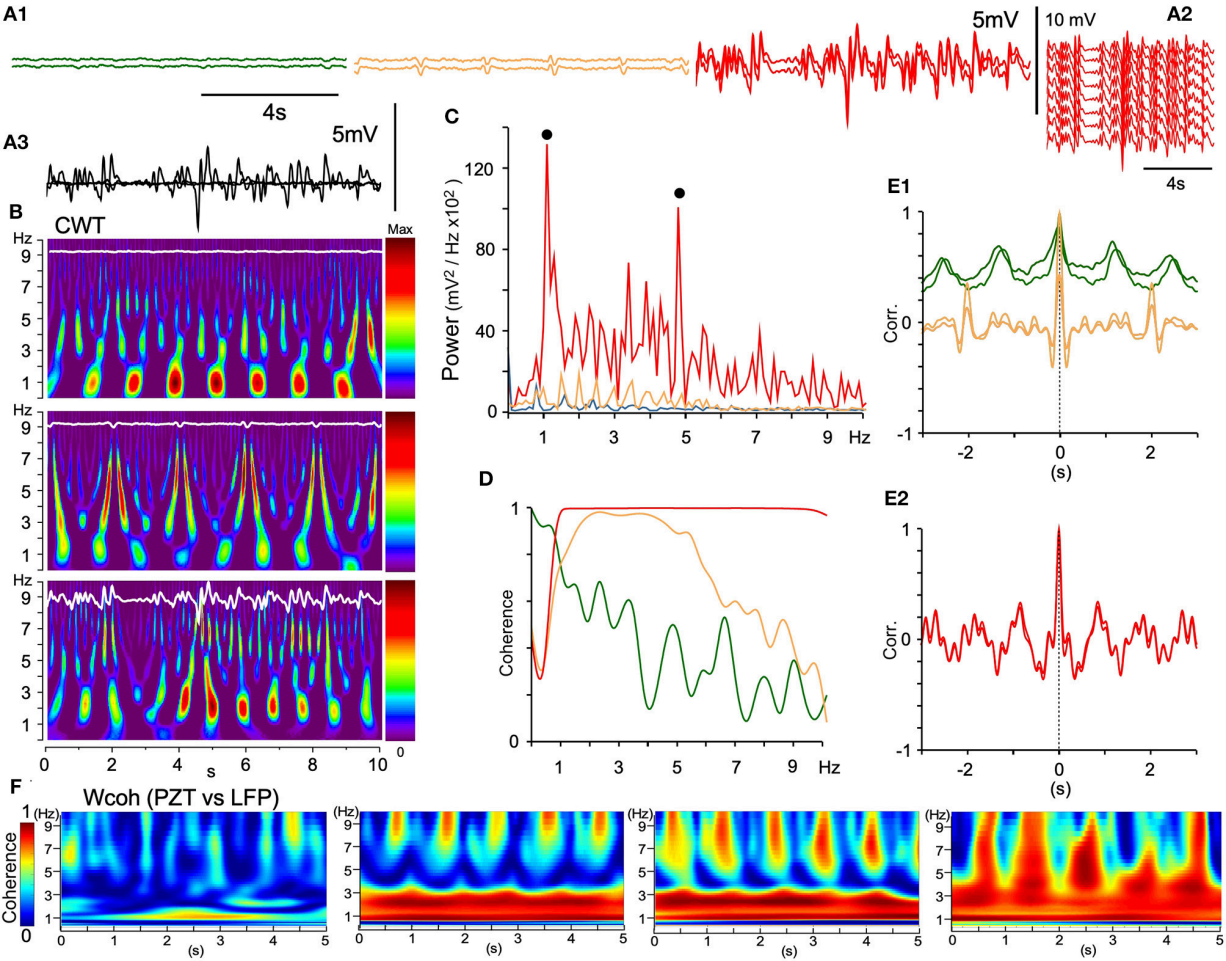
Large-scale spontaneous oscillatory synchrony. **(A1)** Three successive episodes (green, orange, red) of adjacent LFP pairs (50 μm apart) recorded by a silicon multiple electrode. **(A2)** In the third episode, all the LFPs of similar trajectories emerged, ensued, and then disappeared. **(A3)** Superimposition of the episodes. **(B)** Time-resolved power spectra (CWT) of the episodes. **(C)** Power spectra of the episodes (each color same with **A**). Note two power peaks (solid dots; ~1 and ~5 Hz) in the large-scale synchrony (red). **(D)** Coherence spectra of the episodes. **(E1)** and **(E2)**, Superimposition of an autocorrelogram and a correlogram between adjacent paired waves of the three episodes. Note that oscillatory frequencies differ among the episodes and a salient mixture of two oscillation modes (~1 and ~5 Hz) in the last episode **(E2)**. **(F)** Time-resolved coherence spectra (wavelet coherence; Wcoh) between PZT and LFP signals according to an amplitude of LFPs. As LFP amplitudes develop (left to right), dense bands of high coherence occupy a wider range of frequency. PZT, piezoelectric pulse transducer; LFP, local field potential.

In rats anesthetized with ketamine/xylazine followed by isoflurane, every multisite LFP showed different signal forms in terms of wave amplitude and phase in most recording sessions. Eventually local coherence of spatiotemporal dynamics was recognized and analyzed as described above. Occasionally, all 16 channel LFPs in the VC exhibited similar signal forms (Figure 9). Adjacent LFP pairs exhibited local or large-scale coherence with varied wave amplitudes and oscillatory phases (Figures 9A1,3). Fluctuating LFPs were temporally orchestrated into full-fledged oscillatory synchrony in the VC (Figures 9A2,B,C,E2), while exhibiting varied degrees of coherence and correlation with adjacent LFPs (Figures 9D,E1). In local coherence, a ~1 Hz oscillation phase appeared and changed to a ~0.5 Hz phase with a neuronal signal augmented amplitude (Figure 9E1). In large-scale coherence, both ~1 and ~5 Hz phases were conspicuous (Figure 9E2). The full-fledged oscillatory synchrony showed the highest power in two prominent peaks (~1 and ~5 Hz, Figures 9B,C) and full coherence over a ~1–10 Hz range (Figure 9D). Figure 9F shows a development of wavelet coherence (Wcoh) profiles between PZT and LFP signals, indicating a synergistic relationship of LFP amplitude and cardiorespiratory rhythmicity.

## Discussion

In this paper, we have described the structure and dynamics of spontaneous oscillatory synchrony of neuronal activities in the VC with cardiorespiratory rhythmic cycles. It is hypothesized that in addition to cardiorespiratory premotor/motor neurons, many other types neurons in the medulla and pons could exhibit rhythmic synchronous activities, reflecting varied spatiotemporal power spectra of the somatic (respiratory) and autonomic (cardiac) oscillators. The rhythm range coincides to delta (~1–4 Hz) and theta (~4–8 Hz) brain waves that are widely recorded in the brain (Buzsaki, 2006). As a possible ubiquitous phenomenon, functional significance of neuronal oscillatory synchrony in the VC could be highlighted in comparison with several features described extensively in the cerebral cortex, hippocampus, and other connected brain areas (Destexhe et al., 1999; Buzsaki, 2006; Fujisawa and Buzsaki, 2011).

### Neuronal Activities in the Vagal Complex

Extracellular neuronal activities recorded *in vivo* using a typical glass electrode (resistance: ~1–5 MΩ) are usually expressed as either single- or multi-unit spike activities derived from a relatively small number of neurons close to the electrode tip. However, a neuronal population microenvironment close to an electrode tip differs extremely from other brain areas depending on the neuronal size and density. The VC consists mostly of small cells (Yoshioka et al., 2006; Negishi and Kawai, 2011); VC cell size (~10 μm in diameter) and density (~2.0 × 10^5^/mm^3^ in numerical density) would make for a far more numerous and denser cell population near the recording electrode tip than in the cerebral cortex or hippocampus (Buzsaki, 2006; Kawai, 2018), allowing any unusual neuronal activity profiles to be revealed, as demonstrated in the present study. That is, recorded neuronal activities in the VC contained not only typical single- or multi-unit spikes but also longer duration LFP-like waves, and in occasion, high-amplitude potential waves (mostly polyphasic), possibly due to a reflection of synchronized activity produced by the spatially compact neuronal population (Figures 2, 3). Similar results were confirmed by silicon electrode recordings where longer duration LFPs rather than multi-unit spikes were more prominent (Figure 6). The cellular microenvironment of the VC could produce a similar electrical activity profile by electromyogram using a needle electrode rather than a stereotypical profile of neuronal activity as single- or multi-unit spikes (Mills, 2005).

The spatiotemporal dynamics of VC brain waves is very similar to those reported in the cerebral cortex (Destexhe et al., 1999); power spectrum analysis based on recordings of neuronal activities in the VC revealed a presence of fluctuating spontaneous neuronal activity in synchrony with cardiorespiratory rhythms. In the cat cerebral cortex, three typical wave (LFP) patterns (AWAKE, REM: rapid eye movement, SWS: slow wave sleep) were described according to the degree of consciousness, with different spatiotemporal dynamics expressed in terms of wave correlation. LFPs of varied wave amplitudes with similar patterns in not only correlation but also power and coherence were observed to alternatively emerge and disappear in the VC of deeply anesthetized rats.

### Cardiorespiratory Rhythms and Ponto-Medullary Neuronal Circuits

Cardiorespiratory coupling first described by Walter Coleman in 1920 shows several salient features in humans and animals: (1) Coupling becomes more apparent and stable when subjects or animals are sedated or anesthetized. (2) In such occasions, the ratio of heart beats to breaths is adjusted to a whole number. (3) There is an associative interaction between cardiac and respiratory rhythms (Galletly and Larsen, 1997; Dick et al., 2014). For example, in unconscious anesthetized rats, respiratory, and cardiac cycles in the present study were ~1 and ~5 Hz (1:5 ratio), respectively, while in a conscious state the ratio became to 1:2 (~3 vs. ~6 Hz, Kabir et al., 2010).

While the baro-receptor reflex is a vital adjustor of cardiorespiratory coupling, as evidenced in respiratory sinus arrhythmia, it has been claimed that cardiorespiratory synchrony is an expression of another type of cardiorespiratory interaction, such as a central coupling between cardiovascular and respiratory neuronal activities (Schafer et al., 1998; Tzeng et al., 2007). Indeed, the synchrony between VC neuronal activity and cardiorespiratory cycles was confirmed even after a total resection of peripheral sensory inputs.

Cardiorespiratory rhythm reflects the activity of peripheral nerves innervating cardiac and respiratory musculatures (Zhong et al., 1997). Since the neurons of origin are considered to be located in the brainstem and spinal cord, and involved in rhythmogenetic circuits, it is interesting to address whether the rhythmic coupling can be confirmed at the level of single neuronal activities in the brainstem. Indeed, both respiratory and cardiac rhythmic neuronal activities, separately recorded from ponto-medullary neurons, have been extensively analyzed using both *in vitro* and *in vivo* preparations (Feldman and Ellenberger, 1988; Dick et al., 2014). The literature suggests that cardiac or respiratory neuronal populations are mostly separate entities and their coupling may be mediated by a minor population of cell groups, such as rostral ventrolateral medulla (C1) neurons (Guyenet et al., 1990; Montano et al., 1996). On the other hand, there has been, albeit little, evidence demonstrating that neuronal activities at the level of single units contain both cardiac and respiratory cycle frequency components (Boczek-Funcke et al., 1992; Habler and Janig, 1995; Pilowsky, 1995; Ootsuka et al., 2002). The present results add further evidence, raising the possibility of wide-ranging neuronal populations exhibiting both cardiac and respiratory cycle activities in the brainstem.

The dynamics of cardiorespiratory cross-frequency coupling revealed in the present study involving the development of synchrony from fluctuating noisy oscillations might have functional roles related to signal amplification and electrical signal transport to distant regions, rather than serving as passive reflections of neuronal activities resulting from cardiorespiratory rhythmogenesis. The cNTS provides divergent efferent systems up to forebrain regions including catecholaminergic and cholinergic neuronal groups (Kawai, 2018). The parasympathetic preganglionic neurons in the dorsal motor nucleus of the vagus send their axons a great length to reach the abdominal viscera (Ramon y Cajal, 1995). It is tempting to speculate that a strong power produced by oscillatory synchrony may facilitate signal transfer to distant targets.

### Oscillatory Synchrony Across Wide-Range Brain Regions

Large-scale oscillatory synchrony of neuronal activities has been recorded in the cerebral cortex of anesthetized animals and humans during non-REM sleep (Destexhe et al., 1999; Buzsaki, 2006). Spatiotemporal dynamics of oscillation coherence and correlation have been reported to exhibit characteristic behaviors according to different states of consciousness. For example, low-amplitude and low-coherence waves are recorded in an awake state, while high-amplitude and high-coherence slow waves are noted during non-REM sleep. Recent studies show that large-scale oscillatory activities, similar to those recorded from anesthetized animals, are recorded as waves of different phases (4 Hz and theta) in awake animals during task-related behavior (Fujisawa and Buzsaki, 2011). Among the prefrontal cortex, the ventral tegmental area, and the hippocampus, cross-frequency phase coupling (2:1) between 4 Hz and theta oscillators, and joint modulation of local gamma oscillators, has been hypothesized for linking the entorhinal-hippocampal spatial-contextual system with the mesolimbic reward system (Fujisawa and Buzsaki, 2011). The hippocampal theta oscillation could synchronize to establish functional connectivity with the red nucleus for motor behavior adjustment (Del Rio-Bermudez et al., 2017). It is possible that various brain areas can participate in system-wide synchrony within a learning context through theta oscillation. It should be noted that the theta vs. 4 Hz oscillation coupling would be comparable in relation to ~6 vs. ~3 Hz (2:1 ratio) cardiorespiratory coupling in conscious rats (Kabir et al., 2010). The phase-phase (2:1) coupling mechanism might provide a common functional significance, such as a communication link across different brain regions (Canolty and Knight, 2010).

### Emergent Self-Organization

Large-scale collective oscillation seems to emerge spontaneously in the VC possibly due to the cross-frequency coupling. Synchrony is a key concept to the understanding of self-organization phenomena occurring in the fields of coupled oscillators of the dissipative type. Self-organization is observed in natural environments concerned with not only living organisms but various chemical or physical reactions (Kuramoto, 1984). Theoretical description and plausible mechanism of collective generation of high-amplitude bursts of neuronal network have been reported and would help to understand the physiological functions of the VC (Kuramoto, 1984; Fardet et al., 2018). In neuronal networks it is likely that wave amplitude amplification and phase adaptation underlie spontaneous large-scale oscillatory synchrony.

## Conclusions

VC neurons are connected to rhythmogenetic brainstem neuronal circuits that govern salient respiratory (somatic; delta range cycle) and autonomic (theta range cycle) rhythms. Therefore, neuronal activity of the VC could reflect circuit activity of both oscillators in addition to peripheral sensory inputs. However, all or a portion of the two oscillator activities would vary according to consciousness level or quality (such as attention or learning) of animals or individuals. These two brainstem oscillators seem to orchestrate neuronal activities of not only the VC but also other wide-ranging brainstem neuronal groups, including catecholaminergic, cholinergic, and serotonergic systems (Kawai, 2018), through a phase-phase coupling mechanism, to perform specific physiological functions. The oscillatory synchrony and the ascending macrocircuits (Kawai, 2018) could represent functional and anatomic substrates for the presumed ascending reticular activating system including the bulbar reticular formation (Moruzzi and Magoun, 1949). Principles governing the brainstem's life-maintaining function could obey cross-frequency coupling (Canolty and Knight, 2010) and amplitude-death (Zou et al., 2017) theories. Theoretical and computer-simulating investigations would explain a possible basic principle for better understanding a neuronal mechanism of life-maintenance.

## Author Contributions

The author confirms being the sole contributor of this work and has approved it for publication.

### Conflict of Interest Statement

The author declares that the research was conducted in the absence of any commercial or financial relationships that could be construed as a potential conflict of interest.

## References

[B1] Bianchi A. L. Denavit-Saubie M. Champagnat J. (1995). Central control of breathing in mammals: neuronal circuitry, membrane properties, and neurotransmitters. Physiol. Rev. 75, 1–45. 10.1152/physrev.1995.75.1.1 7831394

[B2] Blanche T. J. Spacek M. A. Hetke J. F. Swindale N. V. (2005). Polytrodes: high-density silicon electrode arrays for large-scale multiunit recording. J. Neurophysiol. 93, 2987–3000. 10.1152/jn.01023.2004 15548620

[B3] Boczek-Funcke A. Dembowsky K. Habler H. J. Janig W. Michaelis M. (1992). Respiratory-related activity patterns in preganglionic neurones projecting into the cat cervical sympathetic trunk. J. Physiol. 457, 277–296. 10.1113/jphysiol.1992.sp019378 1297836 PMC1175731

[B4] Buzsaki G. (2006). Rhythms of the Brain. New York, NY: Oxford University Press, Inc 10.1093/acprof:oso/9780195301069.001.0001

[B5] Canolty R. T. Knight R. T. (2010). The functional role of cross-frequency coupling. Trends Cogn. Sci. 14, 506–515. 10.1016/j.tics.2010.09.001 20932795 PMC3359652

[B6] Coleman W. M. (1920). On the correlation of the rate of heart beat, breathing, bodily movement and sensory stimuli. J. Physiol. 54, 213–217. 10.1113/jphysiol.1920.sp001920 16993460 PMC1405248

[B7] Del Rio-Bermudez C. Kim J. Sokoloff G. Blumberg M. S. (2017). Theta oscillations during active sleep synchronize the developing rubro-hippocampal sensorimotor network. Curr. Biol. 27, 1413–1424.e1414. 10.1016/j.cub.2017.03.077 28479324 PMC5446093

[B8] Destexhe A. Contreras D. Steriade M. (1999). Spatiotemporal analysis of local field potentials and unit discharges in cat cerebral cortex during natural wake and sleep states. J. Neurosci. 19, 4595–4608. 10.1523/JNEUROSCI.19-11-04595.1999 10341257 PMC6782626

[B9] Dick T. E. Hsieh Y. H. Dhingra R. R. Baekey D. M. Galan R. F. Wehrwein E.. (2014). Cardiorespiratory coupling: common rhythms in cardiac, sympathetic, and respiratory activities. Prog. Brain Res. 209, 191–205. 10.1016/B978-0-444-63274-6.00010-2 24746049 PMC4052709

[B10] Ezure K. Tanaka I. (1996). Pump neurons of the nucleus of the solitary tract project widely to the medulla. Neurosci. Lett. 215, 123–126. 10.1016/0304-3940(96)12968-2 8888011

[B11] Fardet T. Ballandras M. Bottani S. Metens S. Monceau P. (2018). Understanding the generation of network bursts by adaptive oscillatory neurons. Front. Neurosci. 12:41. 10.3389/fnins.2018.00041 29467607 PMC5808224

[B12] Feldman J. L. Ellenberger H. H. (1988). Central coordination of respiratory and cardiovascular control in mammals. Annu. Rev. Physiol. 50, 593–606. 10.1146/annurev.ph.50.030188.003113 3288108

[B13] Fortin G. Branchereau P. Araneda S. Champagnat J. (1992). Rhythmic activities in the rat solitary complex *in vitro*. Neurosci. Lett. 145, 23–27. 10.1016/0304-3940(92)90194-C 1461562

[B14] Fujisawa S. Buzsaki G. (2011). A 4 Hz oscillation adaptively synchronizes prefrontal, VTA, and hippocampal activities. Neuron 72, 153–165. 10.1016/j.neuron.2011.08.018 21982376 PMC3235795

[B15] Galletly D. C. Larsen P. D. (1997). Cardioventilatory coupling during anaesthesia. Br. J. Anaesth. 79, 35–40. 10.1093/bja/79.1.35 9301386

[B16] Guyenet P. G. Darnall R. A. Riley T. A. (1990). Rostral ventrolateral medulla and sympathorespiratory integration in rats. Am. J. Physiol. 259(5 Pt 2), R1063–1074. 10.1152/ajpregu.1990.259.5.R1063 2173425

[B17] Habler H. J. Janig W. (1995). Coordination of sympathetic and respiratory systems: neurophysiological experiments. Clin. Exp. Hypertens. 17, 223–235. 10.3109/10641969509087067 7735271

[B18] Kabir M. M. Nalivaiko E. Abbott D. Baumert M. (2010). Impact of movement on cardiorespiratory coordination in conscious rats, in Conference Proceedings: Annual International Conference of the IEEE Engineering in Medicine and Biology Society. IEEE Engineering in Medicine and Biology Society. Conference 2010 (Buenos Aires). 1938–1941. 10.1109/IEMBS.2010.5627748 21097002

[B19] Kawai Y. (2018). Differential ascending projections from the male rat caudal nucleus of the tractus solitarius: an interface between local microcircuits and global macrocircuits. Front. Neuroanat. 12:63. 10.3389/fnana.2018.00063 30087599 PMC6066510

[B20] Kuramoto Y. (1984). Chemical Oscillations, Waves, And Turbulence. New York, NY: Dover Publications, Inc 10.1007/978-3-642-69689-3

[B21] Lambertz M. Kluge W. Langhorst P. (1993). Discharge pattern of neurons in the nucleus tractus solitarii (NTS): its cardiac rhythm is modulated by firing rate of the neurons. J Auton. Nerv. Syst. 44, 137–150. 10.1016/0165-1838(93)90026-Q 8227953

[B22] Mills K. R. (2005). The basics of electromyography. J. Neurol. Neurosurg. Psychiatry 76(Suppl. 2), ii32–ii35. 10.1136/jnnp.2005.069211 15961866 PMC1765694

[B23] Miyazaki M. Arata A. Tanaka I. Ezure K. (1998). Activity of rat pump neurons is modulated with central respiratory rhythm. Neurosci. Lett. 249, 61–64. 10.1016/S0304-3940(98)00402-9 9672389

[B24] Miyazaki M. Tanaka I. Ezure K. (1999). Excitatory and inhibitory synaptic inputs shape the discharge pattern of pump neurons of the nucleus tractus solitarii in the rat. Exp. Brain Res. 129, 191–200. 10.1007/s002210050889 10591893

[B25] Montano N. Gnecchi-Ruscone T. Porta A. Lombardi F. Malliani A. Barman S. M. (1996). Presence of vasomotor and respiratory rhythms in the discharge of single medullary neurons involved in the regulation of cardiovascular system. J. Auton. Nerv. Syst. 57, 116–122. 10.1016/0165-1838(95)00113-1 8867094

[B26] Moruzzi G. Magoun H. W. (1949). Brain stem reticular formation and activation of the EEG. Electroencephalogr. Clin. Neurophysiol. 1, 455–473. 10.1016/0013-4694(49)90219-9 18421835

[B27] Negishi Y. Kawai Y. (2011). Geometric and functional architecture of visceral sensory microcircuitry. Brain Struct. Funct. 216, 17–30. 10.1007/s00429-010-0294-5 21153904 PMC3040306

[B28] Ootsuka Y. Rong W. Kishi E. Koganezawa T. Terui N. (2002). Rhythmic activities of the sympatho-excitatory neurons in the medulla of rabbits: neurons controlling cutaneous vasomotion. Auton. Neurosci. 101, 48–59. 10.1016/S1566-0702(02)00181-9 12462359

[B29] Pilowsky P. (1995). Good vibrations? Respiratory rhythms in the central control of blood pressure. Clin. Exp. Pharmacol. Physiol. 22, 594–604. 10.1111/j.1440-1681.1995.tb02072.x 8542669

[B30] Ramon y Cajal S. (1995). Histology of the Nervous System of Man and Vertebrates. New York, NY; Oxford: Oxford Univ Press.

[B31] Sato S. Yamada K. Inagaki N. (2006). System for simultaneously monitoring heart and breathing rate in mice using a piezoelectric transducer. Med. Biol. Eng. Comput. 44, 353–362. 10.1007/s11517-006-0047-z 16937177

[B32] Schafer C. Rosenblum M. G. Kurths J. Abel H. H. (1998). Heartbeat synchronized with ventilation. Nature 392, 239–240. 10.1038/32567 9521318

[B33] Smith J. C. Abdala A. P. Koizumi H. Rybak I. A. Paton J. F. (2007). Spatial and functional architecture of the mammalian brain stem respiratory network: a hierarchy of three oscillatory mechanisms. J. Neurophysiol. 98, 3370–3387. 10.1152/jn.00985.2007 17913982 PMC2225347

[B34] Tzeng Y. C. Larsen P. D. Galletly D. C. (2007). Mechanism of cardioventilatory coupling: insights from cardiac pacing, vagotomy, and sinoaortic denervation in the anesthetized rat. Am. J. Physiol. Heart Circ. Physiol. 292, H1967–H1977. 10.1152/ajpheart.01049.2006 17172271

[B35] Yoshioka M. Okada T. Inoue K. Kawai Y. (2006). Pattern differentiation of excitatory and inhibitory synaptic inputs on distinct neuronal types in the rat caudal nucleus of the tractus solitarius. Neurosci. Res. 55, 300–315. 10.1016/j.neures.2006.04.001 16716422

[B36] Zhong S. Zhou S. Y. Gebber G. L. Barman S. M. (1997). Coupled oscillators account for the slow rhythms in sympathetic nerve discharge and phrenic nerve activity. Am. J. Physiol. 272(4 Pt 2), R1314–R1324. 10.1152/ajpregu.1997.272.4.R1314 9140035

[B37] Zoccal D. B. Furuya W. I. Bassi M. Colombari D. S. Colombari E. (2014). The nucleus of the solitary tract and the coordination of respiratory and sympathetic activities. Front. Physiol. 5:238. 10.3389/fphys.2014.00238 25009507 PMC4070480

[B38] Zou W. Zhan M. Kurths J. (2017). Revoking amplitude and oscillation deaths by low-pass filter in coupled oscillators. Phys. Rev. E 95:062206. 10.1103/PhysRevE.95.062206 28709198

